# Linezolid use in German acute care hospitals: results from two consecutive national point prevalence surveys

**DOI:** 10.1186/s13756-019-0617-0

**Published:** 2019-10-21

**Authors:** Tobias Siegfried Kramer, Frank Schwab, Michael Behnke, Sonja Hansen, Petra Gastmeier, Seven Johannes Sam Aghdassi

**Affiliations:** 1 Charité – Universitätsmedizin Berlin, corporate member of Freie Universität Berlin, Humboldt-Universität zu Berlin, and Berlin Institute of Health, Institute of Hygiene and Environmental Medicine, Berlin, Germany; 2 National Reference Center for Surveillance of Nosocomial Infections, Berlin, Germany

**Keywords:** Linezolid, Antimicrobial use, Antimicrobial stewardship, Surveillance, Point prevalence survey

## Abstract

**Background:**

Linezolid belongs to a reserve group of antibiotics. In recent years, reports on linezolid resistance in gram-positive cocci have become more frequent. Overuse of linezolid is a relevant factor for resistance development. The objective of this study was to describe current prescription practices of linezolid in German hospitals and identify targets for antimicrobial stewardship interventions.

**Methods:**

We analyzed all linezolid prescriptions from the datasets of the consecutive national point prevalence surveys performed in German hospitals in 2011 and 2016. In both surveys, data on healthcare-associated infections and antimicrobial use were collected following the methodology of the European Centre for Disease Prevention and Control.

**Results:**

Overall, the percentage of linezolid among all documented antimicrobials increased significantly from 2011 to 2016 (*p* < 0.01). In 2011, 0.3% (119 of 41,539) patients received linezolid, in 2016 this proportion was significantly higher (0.4%; 255 of 64,412 patients; p < 0.01). In 2016, intensive care units (ICUs) were the wards most frequently prescribing linezolid. The largest proportion of patients receiving linezolid were non-ICU patients. Roughly 38% of linezolid prescriptions were for treatment of skin/soft tissue and respiratory tract infections. In 2016, linezolid was administered parenterally in 70% (*n* = 179) of cases. Multivariable analysis showed that the ward specialty ICU posed an independent risk factor, while Northern and Southwestern regions in Germany were independent protective factors for a high rate of linezolid prescriptions.

**Conclusions:**

In conclusion, we detected potentials for improving linezolid prescription practices in German hospitals. Given the emergence of linezolid resistance, optimization of linezolid use must be a target of future antimicrobial stewardship activities.

## Background

Linzezolid is an antimicrobial substance belonging to the group of oxazolidinones. It is effective against gram-positive cocci, such as staphylococci and enterococci. In the early 2000s, linezolid was introduced into the German market. It was licensed for the treatment of certain bacterial infections caused by methicillin-resistant *Staphylococcus aureus* (MRSA) and vancomycin-resistant enterococci (VRE). These included the treatment of pneumonia caused by MRSA [1–3], as well as severe skin and soft tissue infections [3, 4]. With its classification as a reserve group antibiotic by the World Health Organization, many applications of linezolid have to be regarded as off-label. This especially applies to the treatment of bone and joint infections [5–9], peritonitis [10], bacteremia [11–14], and endocarditis [15]. Additionally, the excellent oral bioavailability opened new opportunities [16].

Resistance against linezolid in gram-positive cocci is caused by a diverse selection of mutations, which have been identified since the introduction of the drug into the market [17]. Cases of linezolid-resistant enterococci and staphylococci have been reported from outbreaks as well as clinical isolates with increasing frequency [18–20]. Resistance against linezolid is also increasing in Germany [21–23]. Prior treatment with linezolid has been identified as a risk factor for linezolid resistance [24]. Given the relevance of this topic and the lack of robust epidemiological data on the subject, the objective of our study was to describe the current practices of linezolid use in German hospitals and to identify targets for antimicrobial stewardship efforts to promote the prudent use of linezolid.

## Methods

Two point prevalence surveys (PPSs) were conducted in acute care hospitals in Germany in the years 2011 and 2016. Data collection in the participating hospitals was executed by trained local hospital staff. For both surveys, training was organized by the German National Reference Center for Surveillance of Nosocomial Infections in special one-day courses to ensure methodological consistency. Per participating hospital, one person had to attend at least one training course. The training comprised of a detailed presentation of the scope and methodology of the survey, and included case vignettes to be completed by all participants. All data were collected in alignment with the methodology and definitions provided by the European Centre for Disease Prevention and Control (ECDC) [25]. In all cases, participation was on a voluntary basis. In the PPSs, data on healthcare-associated infections (HAIs), antimicrobial use, as well as further indicators of infection prevention and control and antimicrobial stewardship, as defined in the ECDC protocol, were collected. Only patients hospitalized at the time of the survey were included. Following a point prevalence approach, only information available at the time of the survey was collected. The specifics of data collection and management have been described elsewhere in more detail [26–28].

From the data gathered, we extracted all linezolid prescriptions, which were recorded by data collectors, for further analysis. Hospitals and wards, which had at least one patient receiving linezolid, were identified and compared to hospitals and wards where linezolid was not used. Analyses were conducted for all hospitals and separately for a core group of 46 hospitals, which took part in both surveys (2011 and 2016). Following the ECDC methodology, every antimicrobial prescription recorded in the survey had to be allocated to an indication. The ECDC protocol differentiated between antimicrobial use for treatment of infections, prophylactic antimicrobial use and antimicrobial use for other or unknown reasons. Treatment was further segregated into treatment for hospital-acquired infections, community-acquired infections and infections acquired in long-term care facilities. We analyzed the indications for linezolid use and if used for treatment of an infection, we described the site of the infection. Furthermore, data on the route of application were collected and analyzed.

As mentioned above, participating hospitals collected data on HAIs and antimicrobial use. However, the two data sets (HAIs and antimicrobial use) cannot be linked without limitations, since definitions for the HAIs did not correspond with definitions for the site of infection in antimicrobial use for treatment. This means, that patients may have documented use of an antimicrobial for treatment of a hospital-acquired infection, but no corresponding HAI was documented, since the ECDC criteria for an active HAI were not fulfilled. In a similar manner, patients with ECDC HAIs may not have a recorded antimicrobial prescription for a hospital-acquired infection. The background is that the indication for antimicrobial use was supposed to reflect the prescribers’ opinion, whereas the ECDC HAIs required the fulfilment of a set of criteria. Despite this difficulty, we analyzed whether a HAI was documented in patients receiving linezolid, and if a pathogen for the HAI was recorded.

Chi-squared test and Mann–Whitney U test were utilized for univariable analysis. Furthermore, we conducted a multivariable logistic regression analysis to identify predictors of a high rate of linezolid use among all antimicrobials in wards with at least one patient receiving linezolid. A high rate was defined as being equal or greater the 75th percentile of all wards with at least one linezolid prescription. To determine this outcome, we performed a multivariable logistic regression analysis by variable selection stepwise forward. The following parameters were included in the model:

- At the hospital level: hospital type; hospital ownership; number of hospital beds; presence of designated staff for antimicrobial stewardship; number of blood cultures per 100 patient days; number of stool tests for *Clostridioides difficile* infection per 100 patient days; participation in a surveillance network for *Clostridioides difficile* infections; participation in a surveillance network for antimicrobial consumption; participation in a surveillance network for antimicrobial resistance; presence of guidelines for antimicrobial use; presence of training for antimicrobial use; presence of bundles for antimicrobial use; presence of checklists for antimicrobial use; presence of audits for antimicrobial use; presence of surveillance of antimicrobial use; presence of feedback of data on antimicrobial use.
- At the ward level: ward specialty; number of ward beds; prevalence of patients with antimicrobial use; percentage of antimicrobials with a reason in notes (i.e. documented indication); presence of a post-prescription review of antimicrobials within 72 h.

All analyses were conducted with SPSS (IBM SPSS statistics, Somer, NY, USA) and OpenEpi (Open Source Epidemiologic Statistics for Public Health, Version. www.OpenEpi.com, updated 2013/04/06, accessed 2019/06/07). A *p*-value of less than 0.05 was considered statistically significant.

### Ethical approval

The German Protection against Infection Act (“*Infektionsschutzgesetz*”) requires all hospitals in Germany to collect data on HAIs and antimicrobial use. Since all data collected were anonymized and handled in accordance with the German Protection against Infection Act, ethical approval and informed consent were not required.

## Results

A total of 132 hospitals took part in the PPS 2011, and 218 hospitals participated in the survey in 2016. The prevalence of patients with antimicrobial use (pooled mean) was 25,5% in 2011 (10,607 of 41,539 patients) and 25.9% in 2016 (16,688 of 64,412 patients). The percentage of patients receiving linezolid increased significantly (*p* < 0.01) from 0.3% (119 of 41,539 patients) in 2011 to 0.4% (255 of 64,412 patients) in 2016. Collectively, the number of linezolid prescriptions among all antimicrobial prescriptions increased significantly (*n* = 119 (0.8%) in 2011 vs. *n* = 255 (1.2%) in 2016; *p* < 0.01). While in 2011 37.1% hospitals (*n* = 49) documented patients with linezolid, this increased to 43.6% (*n* = 95) in 2016. The number of wards which used linezolid significantly increased from 2011 to 2016 (98 vs. 212; p < 0.01). The median percentage of linezolid among all antimicrobial prescriptions in wards with at least one linezolid prescription decreased from 9.5% (interquartile range: 6.5–13.6) to 9.1% (interquartile range: 6.7–14.3). Regional differences in the use of linezolid were detected, with a significant increase in the percentage of participating wards that used linezolid in the West of Germany (Table 1). The majority of wards prescribing Linezolid in 2016 were intensive care units (ICUs) (37.3% (*n* = 79)), surgical non-ICU wards (28.3% (*n* = 60)) and medical non-ICU wards (23.6% (*n* = 50)). The majority of patients receiving linezolid were medical and surgical non-ICU patients. In 2016, roughly 50.6% (*n* = 129) of prescriptions of linezolid were for treatment of hospital-acquired infections. Among all treatments, treatment of skin and soft tissue infections remained a common indication from 2011 (20.2% (*n* = 21)) to 2016 (23.7% (*n* = 55)). Lower respiratory tract infections made up a smaller proportion of documented indications for linezolid in 2016 (18.1% (*n* = 42)) when compared to 2011 (26.0% (*n* = 27)). In 2016, Bone and joint infections (12.1% (*n* = 28)), bacteremia (11.6% (n = 27)) and intraabdominal infections (9.9% (*n* = 23)) represented a large part of the remaining indications for treatment with linezolid (Table 2). On non-ICU wards, 44.2% (*n* = 69) of linezolid was prescribed orally (Table 3).

**Table 1:** Hospitals, wards, and patients with linezolid use. Data from the national point prevalence surveys 2011 and 2016. Data comparison of the two surveys

Parameter	Group	Variable	Number (percentage) or Median (IQR)				p-value
			Total PPS 2011	With linezolid use PPS 2011	Total PPS 2016	With linezolid use PPS 2016	
Participating hospitals	Total		132 (100)	49 (37.1)	218 (100)	95 (43.6)	0.24
	Number of hospital beds	Median (IQR)	359 (182–607)*	607 (410–822)**	305 (186–548)*	519 (310–728)**	0.17*; 0.04**
	Prevalence of patients with antimicrobial use	Median (IQR)	25.1 (19.2–31.2)*	29.5 (25.2–31.9)**	26.2 (19.5–30.5)*	28.7 (20.9–32.9)**	0.67*; 0.59**
	Hospital type	Primary care	71 (100)	13 (18.3)	118 (100)	29 (24.6)	0.32
		Secondary care	28 (100)	18 (64.3)	41 (100)	28 (68.3)	0.73
		Tertiary care	22 (100)	15 (68.2)	36 (100)	30 (83.3)	0.20
		Specialized hospital	10 (100)	2 (20)	23 (100)	8 (34.8)	0.44
		Other/Unknown	1 (100)	1 (100)	0 (0)	0 (n.a.)	n.a.
	Hospital ownership	Public	n.a.	n.a.	103 (100)	57 (55.3)	n.a.
		Private, not for profit	n.a.	n.a.	63 (100)	18 (28.6)	n.a.
		Private, for profit	n.a.	n.a.	31 (100)	11 (35.5)	n.a.
		Other/Unknown	n.a.	n.a.	21 (100)	9 (42.9)	n.a.
Participating wards	Total		2142 (100)	98 (4.6)	3182 (100)	212 (6.7)	**< 0.01**
	Number of ward beds	Median (IQR)	25 (18–34)*	20 (12–33)**	26 (18–34)*	24 (16–34)**	0.01*; 0.08**
	Prevalence of patients with antimicrobial use	Median (IQR)	23.1 (10.0–39.3)*	53.5 (33.3–72.9)**	25.0 (11.5–40.0)*	45.0 (33.3–63.6)**	0.04*; 0.05**
	Percentage of antimicrobials with reason in notes	Median (IQR)	100 (50.0–100)*	95.3 (60.0–100)**	83.3 (44.4–100)*	82.5 (33.8–100)**	< 0.01*; 0.01**
	Percentage of linezolid among all antimicrobials	Median (IQR)	0 (0–0)*	9.5 (6.5–13.6)**	0 (0–0)*	9.1 (6.7–14.3)**	< 0.01*; 0.82**
	Ward specialty	Medical (incl. Geriatrics, neurology)	656 (100)	25 (3.8)	1034 (100)	50 (4.8)	0.32
		Surgical (incl. G/O, urology, ENT)	711 (100)	24 (3.4)	952 (100)	60 (6.3)	**< 0.01**
		ICU	201 (100)	40 (20.0)	346 (100)	79 (22.8)	0.43
		Other/Not specified	545 (100)	9 (1.7)	850 (100)	23 (2.7)	0.20
	Region	West	597 (100)	16 (2.7)	927 (100)	54 (5.8)	**< 0.01**
		North	349 (100)	22 (6.3)	312 (100)	19 (6.1)	0.91
		Southwest	404 (100)	18 (4.5)	813 (100)	57 (7.0)	0.08
		Southeast	250 (100)	17 (6.8)	420 (100)	33 (7.9)	0.62
		East	542 (100)	25 (4.6)	710 (100)	49 (6.9)	0.09
Included patients	Total		41,539 (100)	119 (0.3)	64,412 (100)	255 (0.4)	**< 0.01**
	Patient specialty	Medical (incl. Geriatrics, neurology)	16,276 (100)	37 (0.2)	27,704 (100)	64 (0.2)	0.95
		Surgical (incl. G/O, urology, ENT)	16,828 (100)	39 (0.2)	25,656 (100)	119 (0.5)	**< 0.01**
		Intensive care	1652 (100)	40 (2.4)	2674 (100)	65 (2.4)	0.99
		Other/Not specified	6783 (100)	3 (0.0)	8378 (100)	7 (0.1)	0.37

Where more than one *p*-value per row is given, asterisks are used to indicate the corresponding datasets. *P*-values for variables where median and interquartile range are stated, were calculated with Mann–Whitney U test. *P*-values for variables where number and percentage are stated, were calculated with Chi-squared test. Bold print is used to indicate statistical significance. Abbreviations: *IQR* interquartile range, *PPS* point prevalence survey, *West* North Rhine-Westphalia, *North* Bremen, Hamburg, Lower Saxony, Mecklenburg-West Pomerania, Schleswig-Holstein; *Southwest* Baden-Württemberg, Saarland, Rhineland-Palatinate; *Southeast* Bavaria, Hesse; *East* Berlin, Brandenburg, Saxony, Saxony-Anhalt, Thuringia; *n.a.* not available/not applicable, *FTE* full-time equivalent, *G/O* gynecology and obstetrics, *ENT* otolaryngology, *ICU* intensive care unit

**Table 2:** Indications for linezolid use. Data from the national point prevalence surveys 2011 and 2016. Data comparison of the two surveys

Indication	Variable	Site of infection	Number (percentage)		*p*-value
			PPS 2011	PPS 2016	
All	Total		119 (100)	255 (100)	n.a.
Treatment (CI + LI + HI)	Total		104 (87.4)	232 (91.0)	0.29
	Site of infection (CI+ LI+ HI)	All	104 (100)	232 (100)	
		Bacteremia	10 (9.6)	27 (11.6)	0.38 (a)
		Non-laboratory confirmed systemic infection	9 (8.7)	18 (7.8)	
		Bone/Joint infection	10 (9.6)	28 (12.1)	
		Skin/Soft tissue infection	21 (20.2)	55 (23.7)	
		Intraabdominal infection	5 (4.8)	23 (9.9)	
		Lower respiratory tract infection	27 (26.0)	42 (18.1)	
		Urinary tract infection	4 (3.8)	12 (5.2)	
		Other/Not specified	18 (17.3)	27 (11.6)	
Treatment CI + LI	Total		43 (36.1)	103 (40.4)	0.44
	Site of infection (CI + LI)	All	43 (100)	103 (100)	
		Bacteremia	3 (7.0)	9 (8.7)	0.21 (a)
		Non-laboratory confirmed systemic infection	4 (9.3)	5 (4.9)	
		Bone/Joint infection	4 (9.3)	11 (10.7)	
		Skin/Soft tissue infection	8 (18.6)	28 (27.2)	
		Intraabdominal infection	0 (0)	11 (10.7)	
		Lower respiratory infection	13 (30.2)	19 (18.4)	
		Urinary tract infection	2 (4.7)	6 (5.8)	
		Other/Not specified	9 (20.9)	14 (13.6)	
Treatment HI	Total		61 (51.3)	129 (50.6)	0.90
	Site of infection (HI)	All	61 (100)	129 (100)	
		Bacteremia	7 (11.5)	18 (14.0)	0.94 (a)
		Non-laboratory confirmed systemic infection	5 (8.2)	13 (10.1)	
		Bone/Joint infection	6 (9.8)	17 (13.2)	
		Skin/Soft tissue infection	13 (21.3)	27 (20.9)	
		Intraabdominal infection	5 (8.2)	12 (9.3)	
		Lower respiratory infection	14 (23.0)	23 (17.8)	
		Urinary tract infection	2 (3.3)	6 (4.7)	
		Other/Not specified	9 (14.8)	13 (10.1)	
Other/Unknown			15 (12.6)	23 (9.0)	0.29

Except where specified otherwise, *p*-values were calculated using Chi-squared test. Abbreviations: *PPS* point prevalence survey, *CI* community-acquired infection, *LI* infection acquired in long-term care, *HI* hospital-acquired infection, *n.a.* not applicable; *(a) p-*values were calculated for all listed sites of infection collectively using R by C tables

**Table 3:** Antimicrobial prescriptions and linezolid prescriptions. Data from the national point prevalence surveys 2011 and 2016. Data comparison of the two surveys

Parameter	Variable	Specification	Number (percentage)		*p*-value
			PPS 2011	PPS 2016	
Antimicrobial prescriptions (all)			14,076 (100)	22,086 (100)	n.a.
Linezolid prescriptions (all)			119 (0.8)	255 (1.2)	**< 0.01**
Linezolid prescriptions (all wards)	Total		119 (100)	255 (100)	n.a.
	Dosage	2 × 600 mg	n.a.	227 (89.0)	n.a.
		All other dosages	n.a.	28 (11.0)	
	Route of application	Parenteral	87 (73.1)	179 (70.2)	0.57
		Oral	32 (26.9)	76 (29.8)	
Linezolid prescriptions in non-ICU wards	Total		67 (56.3)	156 (61.2)	n.a.
	Route of application	Parenteral	36 (53.7)	87 (55.8)	0.78
		Oral	31 (46.3)	69 (44.2)	

*P*-values for variables were calculated using Chi-squared test. Bold print is used to indicate statistical significance. Abbreviations: *PPS* point prevalence survey, *ICU* intensive care unit, *n.a.* not available/not applicable

In patients included in the survey 2016 with linezolid use and HAIs as defined by the ECDC, a total of 132 pathogens were documented. Among the most frequently isolated pathogens were enterococci (*n* = 44; 10 of which with resistance against vancomycin), coagulase negative staphylococci (*n* = 21) and *Staphylococcus aureus* (*n* = 19; 12 of which with resistance against methicillin) (Additional file 1: Table S1”). Structural and process parameters of antimicrobial use and antimicrobial stewardship which were only collected in the PPS 2016 (Additional file 1: Table S2), as well as data on a separate analysis of the core group of 46 hospitals participating in both surveys can be also found in the online-supplement (Additional file 1: Table S3) of this article.

Multivariable logistic regression revealed that the ward specialty ICU was significantly associated with a high rate (≥75% percentile) of linezolid prescriptions among all antimicrobial prescriptions in wards with at least one patient receiving linezolid. Conversely, the regions North and Southwest, as well as a 1 % increase in the prevalence of patients with antimicrobial use, were factors significantly decreasing the likelihood of a high rate of linezolid among all antimicrobials. Further parameters, which related to antimicrobial stewardship activities, were not demonstrated to have a significant effect on the rate of linezolid prescriptions (Table 4).

**Table 4:** Multivariable analysis for the outcome high rate of linezolid prescriptions per 100 antimicrobial prescriptions of 212 wards with linezolid use in the point prevalence survey 2016

Outcome	Parameter	Odds ratio	95% confidence intervall	*p*-value
High rate of linezolid prescriptions per 100 antimicrobial prescriptions (≥3Q)	Prevalence of patients with antimicrobial use (per 1% increase)	0.94	0.92–0.96	**< 0.01**
	North (region)	0.19	0.04–0.78	**0.02**
	Southwest (region)	0.43	0.19–0.99	**0.05**
	Intensive care unit (ward specialty)	4.89	2.05–11.70	**< 0.01**

Bold print is used to indicate statistical significance. High was defined as greater or equal than the 75th percentile (3Q). The value for 3Q was 14.3%. Abbreviations: *North* Bremen, Hamburg, Lower Saxony, Mecklenburg-West Pomerania, Schleswig-Holstein; *Southwest* Baden-Württemberg, Saarland, Rhineland-Palatinate

## Discussion

Overall, we did not observe a drastic change in the use of linezolid in German hospitals participating in the two PPSs. Although significantly more patients received linezolid in 2016 than in 2011, the median of the proportion of linezolid among all antimicrobials prescribed in wards with linezolid use slightly decreased, while the 75% percentile increased. This observation could be explained by a few wards with intensified use of linezolid, compared to a larger number of wards trying to restrict linezolid use. Furthermore, a larger proportion of the participating hospitals and wards documented at least one patient receiving linezolid in 2016, when compared to 2011. This development could have been triggered by the reported increase in HAIs caused by VRE in Germany [29]. Observed regional differences in the prevalence of linezolid use might be explained by existing regional differences in the proportion of MRSA and VRE in HAIs in Germany [29, 30].

In 2011, a large number of patients treated with linezolid were hospitalized in ICUs. In 2016, this was still true and could be interpreted as an indication that linezolid is primarily prescribed for treatment of severe infections, such as pneumonia caused by MRSA [31]. However, patients in surgical non-ICU wards were the second most common patient group to receive linezolid in 2016. In this patient group, off-label use of linezolid appears likely [32]. Recommendations by the Surgical Infection Society and the Infectious Diseases Society of America include linezolid as an option in the treatment of complicated intraabdominal infections [33]. However, high quality evidence on this recommendation is scarce. Furthermore, the sole application of international guidelines or guidelines from another country does not consider the underlying epidemiological situation of drug resistance in Germany [34, 35] and therefore, is not always appropriate.

While the above-listed indications and off-label use of linezolid represent one aspect of evaluating linezolid use, dosing and route of application are other important factors. According to our data, linezolid was adequately prescribed as 600 mg twice daily in almost 90% of cases. However, more than half of patients outside of ICUs received linezolid intravenously. This either reflects the severe morbidity of these patients, or shows a lack of knowledge of pharmacokinetics and pharmacodynamics, given the good oral bioavailability of linezolid [16]. Adverse events, especially in patients undergoing parenteral treatment with linezolid for longer than 10 days, have been repeatedly described [36, 37]. Sensitizing prescribers for the adverse effects of linezolid use can be an effective intervention to decrease linezolid use and establish less harmful therapeutic regimens. When this is done, an emphasis should be placed on a multidisciplinary approach at the matter.

Several reports have identified effective antimicrobial stewardship measures focusing on linezolid that did not only decrease use [38] and costs [39], but also led to a reduction of resistance against linezolid [40]. In this context, it is important to acknowledge that despite clinical benefits and cost-effectiveness of linezolid, there are multiple alternative treatment options for multidrug resistant staphylococci infections that were demonstrated to lead to non-inferior outcomes [41], while being even more cost-effective [42].

As shown in our multivariable analysis, the ward specialty ICU represented an independent risk factor for a high rate of linezolid prescriptions. This finding could potentially be explained by the general recommendation to reserve linezolid for the therapy of patients with severe infections and high risk for gram-positive multidrug resistant organisms (e.g. MRSA or VRE). In the case of MRSA, alternative effective treatment options are available and a decrease in methicillin-resistance was described for HAIs caused by *Staphylococcus aureus* in Germany [30]. However, the same does not apply for VRE [29]. Pronounced regional differences were demonstrated in Germany regarding the proportion of vancomycin-resistance in HAIs caused by enterococci. Northern and Southwestern regions of Germany are among the parts of the country with the lowest rates of infections due to VRE [43]. This could represent an explanation for those regions being independent protective factors for a high rate of linezolid use in our multivariable analysis.

### Limitations

Since the national PPSs were not originally designed for linezolid-related analyses, various limitations have to be recognized:

- The data in both surveys were collected mostly by non-prescribers. Therefore, the quality of the collected data is highly dependent on documentation quality by the prescribers and/or interaction with the prescribers. To reduce this confounding effect, the staff collecting data were trained prior to the survey according to the ECDC protocol by members of the German National Center for Surveillance of Nosocomial Infections.
- Participation in the surveys was voluntary. Therefore, centers with a higher motivation to conduct surveillance may be overrepresented. Frequently, these are hospitals with higher rates of HAIs and antimicrobial use. This could conceivably lead to an overestimation with regard to the use of linezolid and other reserve group antibiotics.
- Because of the study design, we were only able to describe pathogens in patients, which fulfilled the requirements for HAIs according to ECDC definitions. In patients with infections, which did not fulfill the ECDC definitions, we cannot make any statement about underlying pathogens. Definitions for the ECDC HAIs did not correspond with definitions for site of infection in antimicrobial use for treatment. Indication for antimicrobial use reflected the prescribers’ opinion, whereas the ECDC HAIs required the fulfilment of a set of criteria. Linkage of the two datasets can only be done with reservations.

## Conclusion

Linezolid is an effective antibacterial substance, but increasing use is associated with resistance. Our data showed that prescription of linezolid is common in German hospitals. Off-label use appears to account for a relevant proportion of prescriptions. Especially surgical non-ICU wards might be a target for antimicrobial stewardship efforts promoting the prudent use of linezolid.

## Supplementary information

**Additional file 1.** Subanalyses and additional data.

## Data Availability

The datasets, on which all analyses are based, are available from the corresponding author upon reasonable request.

## Abbreviations

ECDC: European Centre for Disease Prevention and Control
HAI(s): Healthcare associated infection(s)
ICU(s): Intensive care unit(s)
MRSA: Methicillin-resistant *Staphylococcus aureus*
PPS(s): Point prevalence survey(s)
VRE: Vancomycin-resistant enterococci
