# Supplementary material for: Linezolid use in German acute care hospitals: results from two consecutive national point prevalence surveys
Source: Antimicrob Resist Infect Control. 2019 Oct 21;8:159. doi: 10.1186/s13756-019-0617-0 (PMC6805522; doi:10.1186/s13756-019-0617-0)
Supplement: Supplementary file 1 — Additional file 1. Subanalyses and additional data. [file 13756_2019_617_MOESM1_ESM.pdf]

Table S1 Healthcare-associated infections as defined by the European Centre for Disease Prevention and Control and causing pathogens in patients receiving linezolid. Data from the national point prevalence surveys 2011 and 2016. Data comparison of the two surveys

**Healthcare-associated infection**	**Variable/Pathogen**	**Number**		**p-value**
		**PPS 2011**	**PPS 2016**	
All	Number of infections	67	129	n.a.
	All isolated pathogens	75	132	
	Staphylococcus aureus (MRSA)	14 (9)	19 (12)	
	Enterococcus spp. (VRE)	16 (4)	44 (10)	
	Coagulase-negative staphylococci	10	21	
	Enterobacter spp.	6	6	
	Escherichia coli	7	12	
	Klebsiella spp.	5	6	
	Pseudomonas aeruginosa	3	9	
	Other	14	15	0.43 (a)
Bloodstream infection	Number of infections	9	20	n.a.
	All isolated pathogens	11	28	
	Staphylococcus aureus (MRSA)	2 (1)	6 (4)	
	Enterococcus spp. (VRE)	3 (0)	8 (2)	
	Coagulase-negative staphylococci	3	7	
	Enterobacter spp.	0	0	
	Escherichia coli	2	2	
	Klebsiella spp.	0	1	
	Pseudomonas aeruginosa	0	2	
	Other	1	2	n.a. (a)
Non-laboratory confirmed systemic infection	Number of infections	5	6	n.a.
Lower respiratory tract infection	Number of infections	19	32	n.a.
	All isolated pathogens	21	25	
	Staphylococcus aureus (MRSA)	5 (4)	8 (5)	
	Enterococcus spp. (VRE)	2 (1)	3 (0)	
	Coagulase-negative staphylococci	1	2	
	Enterobacter spp.	2	4	
	Escherichia coli	0	1	
	Klebsiella spp.	2	3	
	Pseudomonas aeruginosa	3	1	
	Other	6	3	0.69 (a)
Surgical site infection	Number of infections	20	33	n.a.
	All isolated pathogens	30	42	
	Staphylococcus aureus (MRSA)	4 (3)	2 (1)	
	Enterococcus spp. (VRE)	7 (3)	20 (5)	
	Coagulase-negative staphylococci	4	6	
	Enterobacter spp.	4	2	
	Escherichia coli	5	5	
	Klebsiella spp.	3	0	
	Pseudomonas aeruginosa	0	3	
	Other	3	4	0.09 (a)
Urinary tract infection	Number of infections	4	11	n.a.
	All isolated pathogens	4	6	
	Staphylococcus aureus (MRSA)	0	0	
	Enterococcus spp. (VRE)	2 (0)	4 (1)	
	Coagulase-negative staphylococci	0	0	
	Enterobacter spp.	0	0	
	Escherichia coli	0	0	
	Klebsiella spp.	0	1	
	Pseudomonas aeruginosa	0	0	
	Other	2	1	n.a. (a)
Clostridioides difficile infection	Number of infections	2	4	n.a.
Other/Not specified	Number of infections	8	23	n.a.

Except where specified otherwise, p-values were calculated using Chi-squared test. Abbreviations: *PPS* point prevalence survey; *MRSA* methicillin-resistant Staphylococcus aureus; *VRE* vancomycin-resistant enterococci; *(a)* p-values were calculated for all listed pathogens collectively using R by C tables

Table S2 Structural and process parameters of antimicrobial use and antimicrobial stewardship of 218 hospitals that participated in the point prevalence survey 2016

**Variable**	**Group/Parameter**	**Number (percentage)** or **Median (interquartile range)**			**p-value**
		**All hospitals (n=218)**	**Hospitals without linezolid use (n=123)**	**Hospitals with linezolid use (n=95)**	
Microbiological diagnostics	Number of blood cultures per 100 patient-days	2.1 (1.3-3.1)	1.7 (0.9-2.6)*	2.5 (1.8-3.5)*	**<0.01***
	Number of stool samples for *Clostridioides difficile* infection per 100 patient-days	0.7 (0.5-1.1)	0.6 (0.2-1.1)*	0.8 (0.6-1.1)*	**0.01***
Surveillance (regional or national network)	*Clostridioides difficile* infection	119 (54.6)	64 (52.0)	55 (57.9)	0.39
	Antimicrobial consumption	83 (38.1)	45 (36.6)	38 (40)	0.60
	Antimicrobial resistance	56 (25.7)	35 (28.5)	21 (22.1)	0.29
Componentes of multimodal strategies (at the hospital level)	Guideline for antimicrobial use	157 (72.0)	90 (73.2)	67 (70.5)	0.67
	Training for antimicrobial use	37 (17.0)	19 (15.4)	18 (18.9)	0.50
	Bundle for antimicrobial use	136 (62.4)	86 (69.9)	50 (52.6)	**<0.01**
	Checklist for antimicrobial use	25 (11.5)	18 (14.6)	7 (7.4)	0.10
	Audit for antimicrobial use	74 (33.9)	44 (35.8)	30 (31.6)	0.52
	Surveillance of antimicrobial use	145 (66.5)	84 (68.3)	61 (64.2)	0.53
	Feedback of data on antimicrobial use	150 (64.2)	82 (66.7)	58 (61.1)	0.40
Post-prescription review of antimicrobials within 72 hours	Percentage of hospital beds	0 (0-16.8)	0 (0-22.4)*	0 (0-15.1)*	0.29*
Designated staff for antimicrobial stewardship	Hospital with designated staff for antimicrobial stewardship	61 (28.0)	35 (28.5)	26 (27.4)	0.86
	Fulltime equivalents per hospital	0 (0-0.1)	0 (0-0.1)*	0 (0-0.2) *	0.80*
	Fulltime equivalents per 250 beds	0 (0-0.1)	0 (0-0.1)*	0 (0-0.1)*	0.58*

Where necessary, asterisks are used to indicate the datasets relating to a p-value. P-values for variables where median and interquartile range are stated, were calculated with Mann–Whitney U test. P-values for variables where number and percentage are stated, were calculated with Chi-squared test. Bold print is used to indicate statistical significance.

Table S3 Antimicrobial use in German acute care hospitals. Data from the national point prevalence surveys 2011 and 2016 of the core group participating in both surveys. Data comparison of the two surveys

**Parameter**	**Group**	**Variable**	**Number (percentage)** or **Median (IQR)**				**p-value**
			**Total PPS 2011**	**With linezolid use PPS 2011**	**Total PPS 2016**	**With linezolid use PPS 2016**	
Participating hospitals	Total		46 (100)	19 (41.3)	46 (100)	22 (47.8)	0.54
	Number of hospital beds	Median (IQR)	368 (261-674)*	666 (434-785)**	392 (229-639)*	578 (383-794)**	0.86*; 0.31**
	Prevalence of patients with antimicrobial use	Median (IQR)	28.0 (20.9-31.8)*	29.9 (26.4-31.7)**	26.5 (20.0-31.3)*	28.6 (20.0-35.9)**	0.82*; 0.92**
	Region	West	16 (100)	4 (25)	16 (100)	3 (18.8)	0.70
		North	6 (100)	1 (16.7)	6 (100)	4 (66.7)	0.13
		Southwest	8 (100)	4 (50)	8 (100)	6 (75)	0.36
		Southeast	9 (100)	5 (55.6)	9 (100)	4 (44.4)	0.67
		East	7 (100)	5 (71.4)	7 (100)	5 (71.4)	>0.99
	Hospital type	Primary care	23 (100)	3 (13.0)	21 (100)	6 (28.6)	0.23
		Secondary care	14 (100)	9 (64.3)	12 (100)	8 (66.7)	0.91
		Tertiary care	8 (100)	7 (87.5)	10 (100)	8 (80)	0.74
		Specialized hospital	1 (100)	0 (0)	3 (100)	0 (0)	n.a.
	Hospital ownership	Public	n.a.	n.a.	21	12 (57.1)	n.a.
		Private, not for profit	n.a.	n.a.	10	3 (30)	n.a.
		Private, for profit	n.a.	n.a.	8	4 (50)	n.a.
		Other/Unknown	n.a.	n.a.	7	3 (42.9)	n.a.
Participating wards	Total		890 (100)	38 (4.3)	868 (100)	50 (5.8)	0.15
	Number of ward beds	Median (IQR)	26 (17-33)*	20 (14-36)**	26 (18-34)*	24 (16-32)**	**0.01***; 0.08**
	Prevalence of patients with antimicrobial use	Median (IQR)	23.7 (10.0-39.2)*	52.2 (33.1-72.5)**	25.0 (12.0-42.3)*	48.8 (38.3-74.0)**	**0.04***; **0.05****
	Percentage of antimicrobials with reason in notes	Median (IQR)	92.3 (50.0-100)*	88.6 (61.1-100)**	83.3 (40.0-100)*	87.0 (34.6-100)	**<0.01***; **0.01****
	Percentage of linezolid among all antimicrobials	Median (IQR)	0.0 (0.0-0.0)*	9.1 (7.1-14.3)**	0.0 (0.0-0.0)*	7.8 (6.2-14.3)**	**<0.01***; 0.82**
	Ward specialty	Medical (incl. geriatrics, neurology)	277 (100)	8 (2.9)	298 (100)	13 (4.4)	0.36
		Surgical (incl. G/O, urology, ENT)	311 (100)	12 (3.9)	277 (100)	14 (5.1)	0.49
		ICU	88 (100)	16 (18.2)	90 (100)	21 (23.3)	0.41
		Other/Not specified	214 (100)	2 (0.9)	203 (100)	2 (1.0)	0.96
	Region	West	249 (100)	9 (3.6)	233 (100)	6 (2.6)	0.53
		North	99 (100)	1 (1.0)	115 (100)	6 (5.2)	0.10
		Southwest	181 (100)	6 (3.3)	162 (100)	14 (8.6)	**0.04**
		Southeast	111 (100)	7 (6.3)	119 (100)	6 (5.0)	0.69
		East	250 (100)	15 (6)	239 (100)	18 (7.5)	0.51
Included patients	Total		17009 (100)	46 (0.3)	17462 (100)	59 (0.3)	0.26
	Patient specialty	Medical (incl. geriatrics, neurology)	6817 (100)	12 (0.2)	7509 (100)	12 (0.2)	0.81
		Surgical (incl. G/O, urology, ENT)	7243 (100)	17 (0.2)	7302 (100)	30 (0.4)	0.06
		Intensive care	801 (100)	17 (2.1)	774 (100)	17 (2.2)	0.92
		Other/Not specified	2148 (100)	0 (0)	1877 (100)	0 (0)	n.a.

Where more than one p-value per row is given, asterisks are used to indicate the corresponding datasets. P-values for variables where median and interquartile range are stated, were calculated with Mann–Whitney U test. P-values for variables where number and percentage are stated, were calculated with Chi-squared test. Bold print is used to indicate statistical significance. Abbreviations: *IQR* interquartile range; *PPS* point prevalence survey; *West* North Rhine-Westphalia; *North* Bremen, Hamburg, Lower Saxony, Mecklenburg-West Pomerania, Schleswig-Holstein; *Southwest* Baden-Württemberg, Saarland, Rhineland-Palatinate; *Southeast* Bavaria, Hesse; *East* Berlin, Brandenburg, Saxony, Saxony-Anhalt, Thuringia; *n.a.* not available/not applicable; *FTE* full-time equivalent; *G/O* gynecology and obstetrics; *ENT* otolaryngology; *ICU* intensive care unit
